# Diagnosis and treatment of CD20 negative B cell lymphomas

**DOI:** 10.1186/s40364-017-0088-5

**Published:** 2017-02-07

**Authors:** Tasleem Katchi, Delong Liu

**Affiliations:** Division of Hematology & Oncology, New York Medical College and Westchester Medical Center, Valhalla, NY 10595 USA

## Abstract

CD20 negative B cell non-Hodgkin lymphoma (NHL) is rare and accounts for approximately 1-2% of B cell lymphomas. CD20- negative NHL is frequently associated with extranodal involvement, atypical morphology, aggressive clinical behaviour, resistance to standard chemotherapy and poor prognosis. The most common types of these include plasmablastic lymphoma, primary effusion lymphoma, large B-cell lymphoma arising from HHV8-associated multicentric Castleman’s disease, and ALK+ large B cell lymphoma. This review provides an overview of the diagnostic and treatment modalities for CD20 negative B cell NHL.

## Background

CD20 is a glycosylated phosphoprotein expressed on the surface of all B cells (except early pro-B cells and plasma cells). Human CD20 molecule is encoded by the MS4A1 gene located on chromosome 11q12.2 [1, 2]. CD20 molecule is a tetra-transmembrane polypeptide with 297 amino acid residues. It plays a role in the differentiation, maturation and activation of B cells. CD20 is involved in the phosphorylation cascade of intracellular proteins by binding to Src family tyrosine kinases, such as Lyn, Fyn, and Lck. The CD20 molecule remains on the membrane of B cells without dissociation or internalization upon binding of CD20 antibody. CD20 expression varies in different lymphoma subtypes [3–5]. It is present from late pro-B cells through memory B cells, but not on early pro-B cells, plasmablasts and plasma cells. Plasma cell differentiation of B cells results in acquisition of plasma cell markers and loss of B cell antigens including the expression of CD20. CD20 was first defined by the murine monoclonal antibody (MoAb) tositumomab [6, 7]. Rituximab, a chimeric CD20 MoAb, was later developed and approved for treatment of human B cell malignancies. Rituximab destroys B lymphoid malignancies through complement-dependent cytotoxicity (CDC) and antibody-dependent cellular cytotoxicity (ADCC). The addition of rituximab, to cyclophosphamide, doxorubicin, vincristine, and prednisone (CHOP) has dramatically improved the survival of patients with diffuse large B cell lymphoma (DLBCL) [8, 9]. R-CHOP has since become the gold standard for the treatment of newly diagnosed DLBCL. In addition, rituximab has been found highly effective in a variety of B cell malignancies as well as relapsed and refractory lymphomas. Through recombinant DNA technology, second- and third- generation CD20 MoAbs were developed [2]. Among these, ofatumumab and obinutuzumab have been approved for clinical treatment of B cell malignancies, such as chronic lymphoid leukemia, and follicular lymphoma [10–15].

Genetic mutations of MS4A1 leading to conformational changes in the protein have been speculated to be a molecular mechanism of the CD20 negative phenotype [16]. The loss of CD20 expression is associated with extranodal involvement, a more aggressive clinical course, loss of responsiveness to rituximab and conventional chemotherapy, leading to poor prognosis. It poses a diagnostic and therapeutic dilemma and further studies need to be undertaken to establish the standard of care in this group of patients.

### CD20 negative non-Hodgkin lymphomas

The pan-B lymphocyte markers include CD19, CD20, CD79a, and PAX-5 [2, 17–19]. Almost all B cell NHLs are positive for CD20. CD20- negative NHLs are rare with a rate of 1–2% of all B cell NHLs [20]. The most common types of these include plasmablastic lymphoma, primary effusion lymphoma, large B-cell lymphoma arising from HHV8-associated multicentric Castleman’s disease, and ALK+ large B cell lymphoma [20, 21].

Plasmablastic lymphoma (PBL) is the most common subtype of CD20 negative DLBCL, accounting for 75% of the cases with a median survival of 12 months [22, 23]. PBL is frequently associated with HIV and/or Epstein-Barr virus (EBV) co-infection. Immunoblastic lymphoma is frequently related and can be difficult to differentiate from PBL.

Primary effusion lymphoma (PEL), as the name suggests, presents as pleural, peritoneal and/or pericardial effusion. It is associated with HIV, EBV, and human herpesvirus 8 (HHV8) co-infection and has a median survival of 9 months [24].

Large B-cell lymphoma arising from HHV8-associated multicentric Castleman disease (MCD) usually presents in the setting of HIV infection. Unlike HHV-8 associated PEL, large B-cell lymphomas arising from MCD frequently has unmutated immunoglobulin IgM and lambda-chain restriction, suggesting an origin from HHV-8- positive plasmablasts [25].

Anaplastic lymphoma kinase (ALK) -positive DLBCL is a very rare type of DLBCL [26]. Unlike ALK+ anaplastic large cell lymphoma which harbors the ALK-NPM fusion gene from t(2;5) translocation with favorable prognosis, ALK+ DLBCL usually has t(2;17) (p23; q23) translocation which leads to a fusion gene of ALK-CLTC [27, 28]. Unlike the common DLBCL, ALK+ DLBCL is usually positive for CD38, CD138, and negative for CD20, CD30, and CD79a [28]. This type of lymphoma has a median survival of 20 months.

In addition to the above rare CD20 negative lymphomas, CD20 positive lymphoma can relapse as CD20 negative lymphoma after CD20 antibody therapy [29].

### Diagnosis of CD20 negative NHL

DLBCL is identified by morphology and B cell biomarker analysis by immunohistochemistry and flow cytometry studies. However, CD20 negative DLBCL can pose a diagnostic dilemma. Immunohistochemical detection of CD19, CD79a and PAX-5 are the major biomarkers in establishing the diagnosis of CD20 negative B cell lymphoma. CD5 expression in DLBCL is mostly associated with Richter’s transformation from a low-grade B-cell lymphoma, but has been seen in 5% of de novo DLBCL [30]. Similarly, CD10 expression is seen in both de novo DLBCL as well as in transformed follicular lymphomas [31]. Oct-2, Bob-1, and SOX11 are frequently examined and useful for differential diagnosis and accurate classification of lymphoma diagnosis [32, 33]. Flow cytometric analysis can reveal positivity for CD19, CD79a, CD5 and CD10 in cases of CD20 negative lymphoma.

Molecular analysis using cytogenetics or FISH (fluorescent in-situ hybridization) to detect rearrangements or translocations of Bcl-2, Bcl-6 and MYC is an important part of diagnosis. BCL-2 mutation was found frequently in human B cell lymphomas [34, 35]. Rearrangements or translocations of both BCL-2 and MYC are hallmarks of “double-hit” lymphomas which are typically more resistant to R-CHOP and portent poor prognosis. More intensive chemotherapy regimens and new agents like ibrutinib and lenalidomide appear to improve responses in these double-hit lymphomas [36].

#### Treatment strategies

There is still no standard of care for CD20 negative B cell lymphomas. Response to standard CHOP chemotherapy is inadequate. CODOX-M/IVAC (cyclophosphamide, vincristine, doxorubicin, methotrexate alternating with ifosfamide, etoposide, cytarabine) [37–44], dose-adjusted EPOCH (infusional etoposide, vincristine and doxorubicin along with bolus cyclophosphamide and prednisone) [45–47], and HyperCVAD (cyclophosphamide, vincristine, doxorubicin and dexamethasone alternating with high-dose methotrexate and cytarabine) [48–52], are the suggested therapies. Upregulation of the expression of CD20 in CD20-negative B cell acute lymphoblastic leukemia following treatment with 5-azacytidine has been reported [53]. In addition, good response to bortezomib in combination with infusional dose-adjusted EPOCH for the treatment of plasmablastic lymphoma has also been reported [54]. Upregulation of CD20 expression by epigenetic agents may be another option to re-sensitize B lymphoma to CD20 antibodies [55]. Plerixafor, a CXCR4 antagonist, has been shown to enhance rituximab-induced killing of lymphoma cells [56]. It would be interesting to examine whether plerixafor can have similar effect in CD20 negative lymphomas.

## Conclusion

CD20 negative lymphoma is uncommon and has poor prognosis. It poses a diagnostic and therapeutic dilemma. Further studies need to be undertaken to establish the standard of care for this group of patients. Novel agents targeting B cell signalling pathways, such as, inhibitors of Bruton tyrosine kinase and phosphoinositol-3 kinase, may play important role in the therapy of this rare entity of B cell lymphomas [57–62]. PD-1 antibodies are active in lymphomas [63–65], it remains important to evaluate whether immune check point inhibitors have activity in CD20 negative lymphomas. Bcl-2 inhibitors may be another option for CD20 negative lymphomas and warrant further investigations [34, 66, 67].

## Abbreviations

CHOP: cyclophosphamide, doxorubicin, vincristine, and prednisone
CODOX-M/IVAC: cyclophosphamide, vincristine, doxorubicin, methotrexate alternating with ifosfamide, etoposide, cytarabine
EPOCH: etoposide, vincristine and doxorubicin along with bolus cyclophosphamide and prednisone
HyperCVAD: cyclophosphamide, vincristine, doxorubicin and dexamethasone alternating with high-dose methotrexate and cytarabine
MoAb: monoclonal antibody
